# Loss of tumor-infiltrating lymphocytes and poor response to immunotherapy in *IDH* GOF mutant melanoma

**DOI:** 10.1172/jci.insight.195384

**Published:** 2026-04-09

**Authors:** Emma Specht, Lakshmi Pakanati, Meng-Ju Wu, Russell W. Jenkins, Derek N. Effiom, Nabeel Bardeesy, Bradley E. Bernstein, Moshe Sade-Feldman, Christine G. Lian, Genevieve M. Boland, Elena Torlai Triglia, Sonia Cohen

**Affiliations:** 1 Department of Surgery, Massachusetts General Hospital, Harvard Medical School, Boston, Massachusetts, USA.; 2 Krantz Family Center for Cancer Research, Department of Medicine, Massachusetts General Hospital, Boston, Massachusetts, USA.; 3 Broad Institute of MIT and Harvard, Cambridge, Massachusetts, USA.; 4 Division of Gastroenterology, Department of Medicine, University of Massachusetts Chan Medical School, Worcester, Massachusetts, USA.; 5 Division of Medical Oncology, Massachusetts General Hospital, Boston, Massachusetts, USA.; 6 Department of Cancer Biology, Dana-Farber Cancer Institute, Boston, Massachusetts, USA.; 7 Departments of Cell Biology and; 8 Department of Pathology, Brigham and Women’s Hospital, Harvard Medical School, Boston, Massachusetts, USA.; 9 School of Biological and Behavioural Sciences, Queen Mary University of London, London, United Kingdom.

**Keywords:** Genetics, Immunology, Oncology, Cancer immunotherapy, Epigenetics, Melanoma

## Abstract

Recent innovations in melanoma treatment with immune checkpoint blockade (ICB) have improved overall outcomes for patients; however, over 50% of patients still develop resistance to treatment. These patients either have intrinsic resistance and never respond to therapy or develop acquired resistance months or years into treatment. The mechanisms underlying ICB resistance remain poorly understood. Our data show that patients with isocitrate dehydrogenase gain-of-function (*IDH* GOF) mutant melanoma have a worse response to anti-PD1 immunotherapy. *IDH* mutations have been found to be oncogenic and associated with differential methylation in multiple cancers but are not yet characterized in human melanoma. Here, we investigate the clinical, immune, and transcriptional phenotypes of *IDH* GOF melanomas through analyses of clinical response, single-cell RNA-seq, bulk RNA-seq, and DNA methylation data. Single-cell data analysis showed decreased immune infiltrate and activity in the *IDH* GOF tumors. Bulk sequencing data demonstrated the association among *IDH* mutation, immune exclusion, and disruptions in global DNA methylation. The melanoma-derived genomic data presented support previously described resistance mechanisms of *IDH* mutation in other cancer types and is the first demonstration to our knowledge of the role of *IDH* GOF in the human melanoma tumor microenvironment.

## Abstract 2

Mutations of the IDH enzyme in melanoma lead to gene silencing and decreased immune function, ultimately leading to a worse response to immunotherapy.

## Introduction

Immunotherapy, including immune checkpoint blockade (ICB) and tumor-infiltrating lymphocyte (TIL) therapy, has become the standard of care for the treatment of advanced melanoma. Unfortunately, the majority of patients demonstrate resistance to treatment with single-agent regimens — up to 56% of patients treated with nivolumab are nonresponders to therapy at 5 years (1). Even with novel combination therapies, over 50% of patients with melanoma have de novo or acquired resistance to therapy and ultimately die of their disease (2). Those with intrinsic resistance to single-agent immunotherapy show limited response at 6 months (3). Other patients with acquired resistance will show an initial favorable response to therapy but later progress. An increased understanding of these resistance mechanisms and their link to genetic alterations will allow for better outcomes for patients with melanoma through tailored therapeutic approaches and rapid identification of resistant patients.

The majority of cutaneous melanoma is driven by UV exposure, which leads to an overall high mutational burden and complicates the identification of driver mutations (4). In a large tumor sequencing effort, The Cancer Genome Atlas (TCGA) program identified 15 gene mutations considered to be significantly present above the background mutation rate in melanoma samples (5). Among these suggested driver mutations, gain-of-function isocitrate dehydrogenase 1 (*IDH1* GOF) mutations with alterations to the R132 site were present in 5%–7% of melanoma tumors. This mutation has been characterized as oncogenic in several other cancers, including cholangiocarcinoma, acute myeloid leukemia, and glioma (6). In its nonmutated form, *IDH* is part of the KREB metabolic cycle and converts isocitrate to α-ketoglutarate through the conversion of NADP^+^ to NADPH. The GOF form of *IDH* converts α-ketoglutarate into the oncometabolite D-2-hydroxyglutarate (D-2-HG). D-2-HG has been shown to alter cell differentiation state, disrupt epigenetic modifications such as DNA methylation, and affect immune function (6, 7). In TCGA analysis, *IDH* GOF melanoma was enriched among the 20% of melanomas with the highest levels of DNA methylation, suggesting that understanding the role of *IDH* GOF mutation in melanoma may provide insight into a large proportion of melanomas.

To study the role of *IDH* GOF in melanoma, we developed a curated cohort of all patients with *IDH* GOF mutations (R132 mutants). All patients with melanoma being considered for systemic therapy at our institution undergo a panel of next-generation sequencing (NGS) of their tumor for at least 50 oncogenic variants (8). Many of these patients also belong to our institutional biobank with tissue samples obtained throughout their treatment course. Using the NGS panel we were able to identify patients with *IDH* GOF mutant melanomas. For these *IDH* GOF patients, we collected clinical features, including histological features of the patients’ primary tumors and demographic details, and characterized their response to any treatments they received. We then profiled the tumors and immune system of *IDH* GOF melanomas. We analyzed tumor and stromal gene expression profiles (GEPs) from a subset of these melanomas at single-cell resolution, which we integrated with published bulk RNA-seq and methylation array data.

Our analysis revealed that patients with *IDH* GOF melanoma have a worse response to anti-*PD1* therapy in the metastatic setting than well-matched *IDH* WT patients, with features suggestive of intrinsic resistance.

The *IDH* GOF tumor samples had fewer immune cells, and of those immune cells present, fewer were functional CD8^+^ T cells. Transcriptional profiles of the tumor cells revealed a significant decrease in global gene expression, including *IFN* response and viral mimicry pathways, with higher methylation of the downregulated genes in *IDH* GOF tumors. Together our findings suggest that *IDH* GOF mutation in melanoma predicts a poor response to anti-*PD1* ICB due to cell-intrinsic alterations in transcriptional programs and consequent immune exclusion.

## Results

### Individuals with IDH GOF mutant melanoma respond poorly to anti-PD1 therapy.

**Figure 1:**
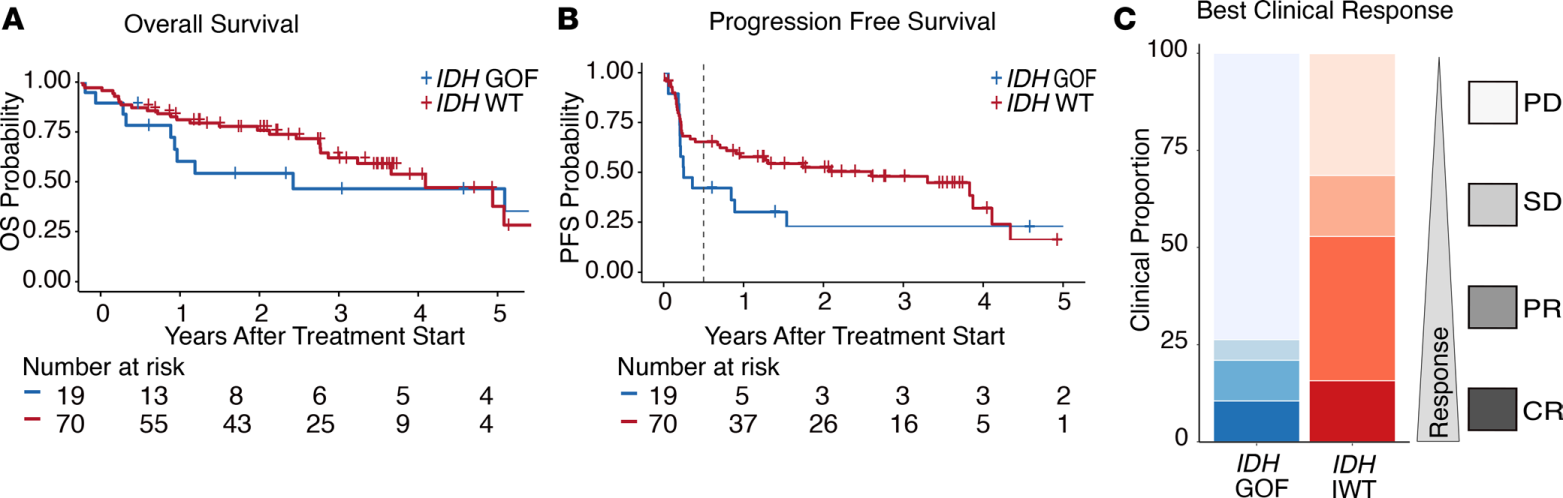
Intrinsic resistance to anti-*PD1* therapy in patients with metastatic *IDH* GOF melanoma. (**A** and **B**) Kaplan-Meier survival analysis of (**A**) overall survival (OS) and (**B**) progression-free survival (PFS) of patients with melanoma treated with anti-*PD1* therapy in the metastatic setting suggests worse outcomes in patients with *IDH* GOF mutant melanoma (blue line) compared with patients lacking the mutation (red line). This separation in progression-free survival begins before the first year of response, suggestive of intrinsic resistance (**B**, dotted line). (**C**) Comparing patients’ best response to immunotherapy (as defined by the treating clinician), mutant patients were more likely to be nonresponders (*P* = 0.004). Dark to light colors represent a gradient from strong to absent response. CR, complete response; PR, partial response; SD, stable disease; PD, progressive disease.

To investigate whether *IDH* GOF mutation affected the clinical responses to immunotherapy in melanoma, we performed institutional retrospective chart review of 823 total patients with melanoma diagnosed between 2010 and 2023 (Supplemental Figure 1; supplemental material available online with this article; https://doi.org/10.1172/jci.insight.195384DS1). 44 total patients were identified with a *IDH1* or *IDH2* GOF mutation from our institutional tumor NGS genetic testing tool (8). This number represents about 6% of all patients tested, in line with published estimates of the prevalence of *IDH* GOF mutations in melanoma (5). Patients with *IDH* WT melanoma were identified by the same NGS testing and were selected to have no statistically significant differences in histopathologic and demographic features compared with the control cohort (Table 1). Of those 44 *IDH* GOF patients, we analyzed the 19 patients (17 patients with an *IDH1* GOF, and 2 patients with an *IDH2* GOF) who received first-line anti-PD1 therapy in the metastatic setting. Looking at common comutations in melanoma more broadly, the abundance of *BRAF* V600 in the 19 *IDH* GOF patients was 26.4% (Table 1) and the presence of *KIT* mutations was 0%. We measured 2 time points relative to the start of therapy: the date of first tumor progression after commencement of therapy and the date of death or last follow-up. The *IDH* GOF patients tended toward worse overall survival and progression-free survival than WT patients, especially in the first 3 years after treatment (Figure 1, A and B). Previous work defined intrinsic resistance to anti-*PD1* as resistance in the first 6 months of treatment (3). Following this definition, our separation in PFS and OS at the 6-month mark suggests intrinsic resistance to anti-*PD1* in *IDH* GOF melanoma. We also investigated differences in best clinical response to therapy, defined as the best treating-physician-determined response to therapy. We called “responders” patients who had a period of either complete or partial response following commencement of therapy. “Nonresponders” were instead patients who had progressive or stable disease as their best response. Consistent with our previous observations on survival, *IDH* GOF patients showed a significantly higher proportion of nonresponders in comparison to the WT cohort (Figure 1C). Taken together, these results suggested that *IDH* GOF mutations lead to intrinsic resistance to anti-*PD1* immunotherapy.

### Mutant IDH tumors have decreased tumor infiltrate and more immunosuppressive T cell features compared with WT tumors.

**Figure 2:**
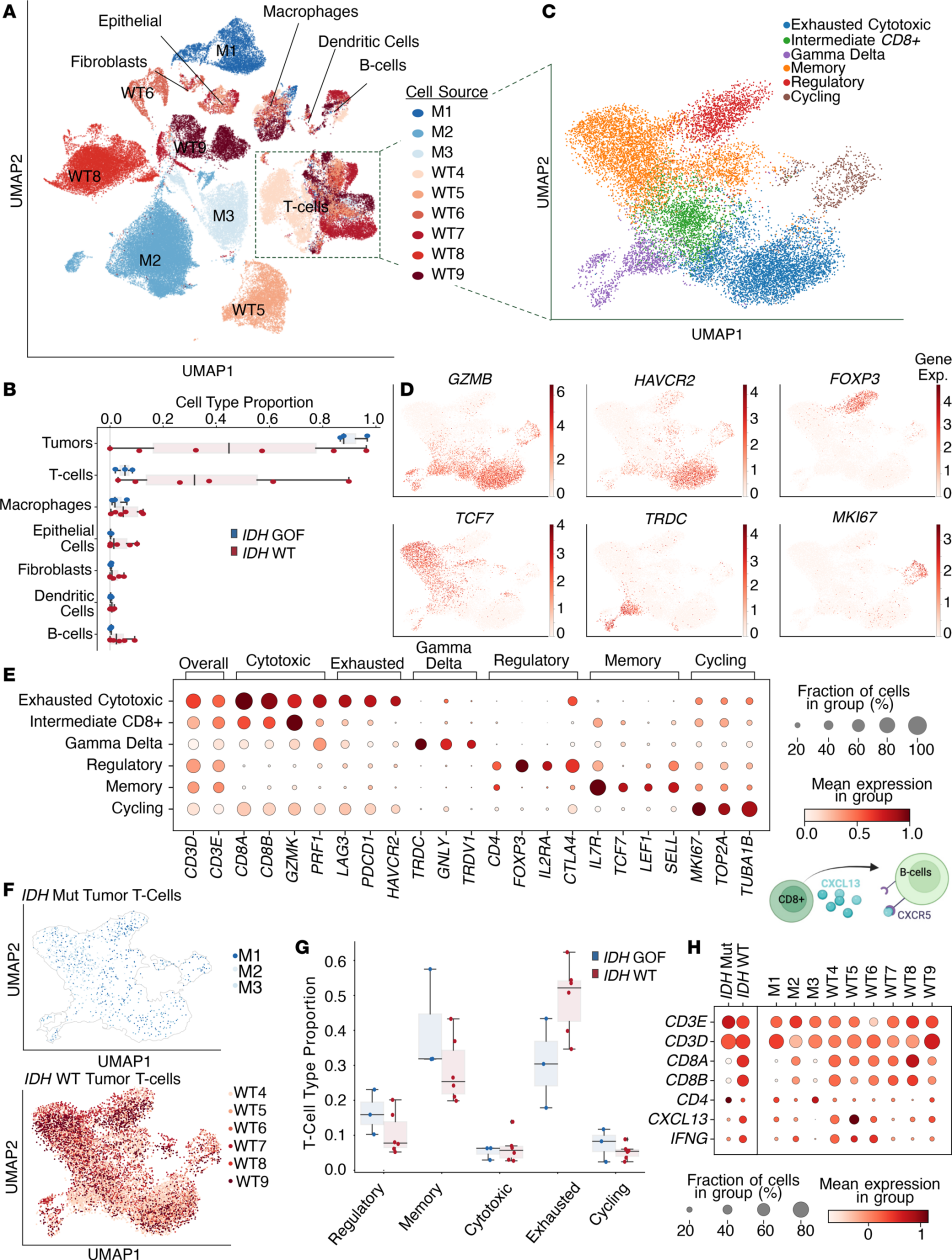
Single-cell RNA-seq shows a decrease in TIL in *IDH* GOF melanoma. Single-cell gene expression profiles from *IDH* GOF melanoma (*n* = 3 patients; 33,076 total high-quality cells; 6,566–20,786 cells per patient) were compared with profiles from well-matched *IDH* WT melanoma (*n* = 6 patients; 38,850 total high-quality cells; 4,129–10,641 per patient) in the pretreatment setting (Supplemental Figure 2). Labels M1–M3 refer to samples from *IDH* GOF patients. Labels WT4–WT9 refer to samples from *IDH* WT patients. (**A**) Cell gene-expression profiles (dots) were annotated by cell types (clusters) and colored by the mutational status of the tumors. Patient tumor samples were analyzed and cell types classified using Leiden clustering in conjunction with marker genes (Methods). (**B**) Box plot of cell type distribution in *IDH* GOF vs. *IDH* WT melanoma with dots representing individual melanomas. (**A** and **B**) Analysis with scCODA revealed that *IDH* GOF tumors had a larger proportion of tumor cells. (**D**) UMAPs representing the expression of marker genes show the normalized expression (red). (**E**) Dot plot showing expression of marker genes in classified T cell type groups. (**D** and **E**) Gene expression profile analysis with Hotspot on all T cells demonstrated 6 modules corresponding to T cell states. (**C**) Cells were assigned to their top module, and genes in each module were used to identify the modules identity (Methods). (**D** and **E**) Well-defined modules of T cell state exhibit distinctly higher expression of state-defining marker genes compared with other modules. (**F**) Mutant tumors had low proportions of T cells compared with WT tumors. (**G**) Comparing the *IDH* GOF and *IDH* WT samples, on average mutant samples included proportionally more regulatory and memory T cells and fewer *CD8*^+^ cytotoxic and exhausted T cells, representing a cold environment. (**H**) T cells from *IDH* WT tumors displayed higher expression of the *CXCL13/CXCR5* signaling pathway, consistent with published work (12) understanding ICB melanoma responders.

To gain insights into the possible molecular mechanism behind resistance to immunotherapy, we sought to understand the cell type composition and transcriptome of these *IDH* GOF tumors. Within the larger clinical cohort of patients with *IDH* GOF mutant melanoma, we were able to generate single-cell RNA-seq (scRNA-seq) profiles from 3 tumor samples from *IDH* GOF tumors and 6 matched *IDH* WT control tumor samples. All samples were from metastasis sites and collected before treatment. We individually matched the *IDH* GOF samples to control samples based on treatment setting, site, stage at time of sample, primary type, sex, and age, in that order (Supplemental Figure 2). Samples were then individually quality controlled to filter for low count genes, low count cells, ambient RNA, doublets, and debris (Methods), obtaining 71,926 high-quality cell profiles. We annotated each GEP using Leiden clustering and known marker genes (Methods, Figure 2A, and Supplemental Figure 3). The *IDH* GOF samples had a very different cell type composition compared with WT samples: GOF samples were composed of large tumor cell clusters with only a very small fraction of immune and epithelial clusters. Statistical testing through scCODA (9) showed that *IDH* GOF tumors had a significantly higher proportion of tumor cells than *IDH* WT samples (fibroblasts were used as a reference cell type).

Moreover, the *IDH* GOF samples were very similar to one another in terms of cell type composition, while the *IDH* WT samples were more heterogeneous (Figure 2B). This finding that *IDH* GOF tumors were primarily made up of tumor cells was supported by purity estimation in the larger TCGA cohort that found *IDH* GOF samples were on average more “pure” tumors (Supplemental Figure 5). Importantly, low levels of immune cells within the *IDH* GOF tumors may explain the lack of immunotherapy response (10).

Using Hotspot to identify groups of genes with correlated expression across single cells (11), we explored the GEP of T cells from *IDH* GOF and *IDH* WT tumors. We identified 6 GEP modules that corresponded to 5 major T cell states: regulatory, memory, cycling, cytotoxic, and exhausted. These modules were supported by both manual clustering as well as using known gene markers to perform cell expression scoring (Figure 2C and Supplemental Figure 3). We classified every cell by the GEP that it most highly used and found that, in tumors from *IDH* GOF patients, T cells were much more likely to have a regulatory and memory phenotype than T cells derived from *IDH* WT tumors (Figure 2G). Additionally, the *IDH* GOF tumors included fewer *CD8^+^* cytotoxic and *CD8*^+^ exhausted cells, pointing to a relatively immunosuppressive environment in *IDH* GOF tumors relative to *IDH* WT tumors.

Previously published work found that the *CXCL13*/*CXCR5* signaling axis between T cells and B cells distinguished responders and nonresponders to anti-*PD1* therapy in melanoma (12). As increased expression of *CXCL13* and *INFG* was found to be positively correlated with response to immunotherapy (12), we investigated the expression of these genes in *IDH* GOF tumors. Consistent with poor response to anti-*PD1* therapy in melanoma, *CXCL13* and *IFNG* expression was low in T cells derived from the *IDH* GOF tumors (Figure 2H). This suggests that *IDH* GOF melanoma–derived T cells are similar to T cells from other melanomas with poor response to anti-*PD1*.

These analyses of tumor GEPs suggest that *IDH* GOF melanomas have a high proportion of tumor cells (with a smaller T cell infiltrate), compared with *IDH* WT melanomas. Moreover, the GEPs of TILs present in *IDH* GOF melanoma are consistent with the poor clinical responses seen to anti-*PD1* therapy in patients with *IDH* GOF melanoma.

### Ligand-receptor interaction and spatial imaging analysis predict less active T cells in IDH TME.

**Figure 3:**
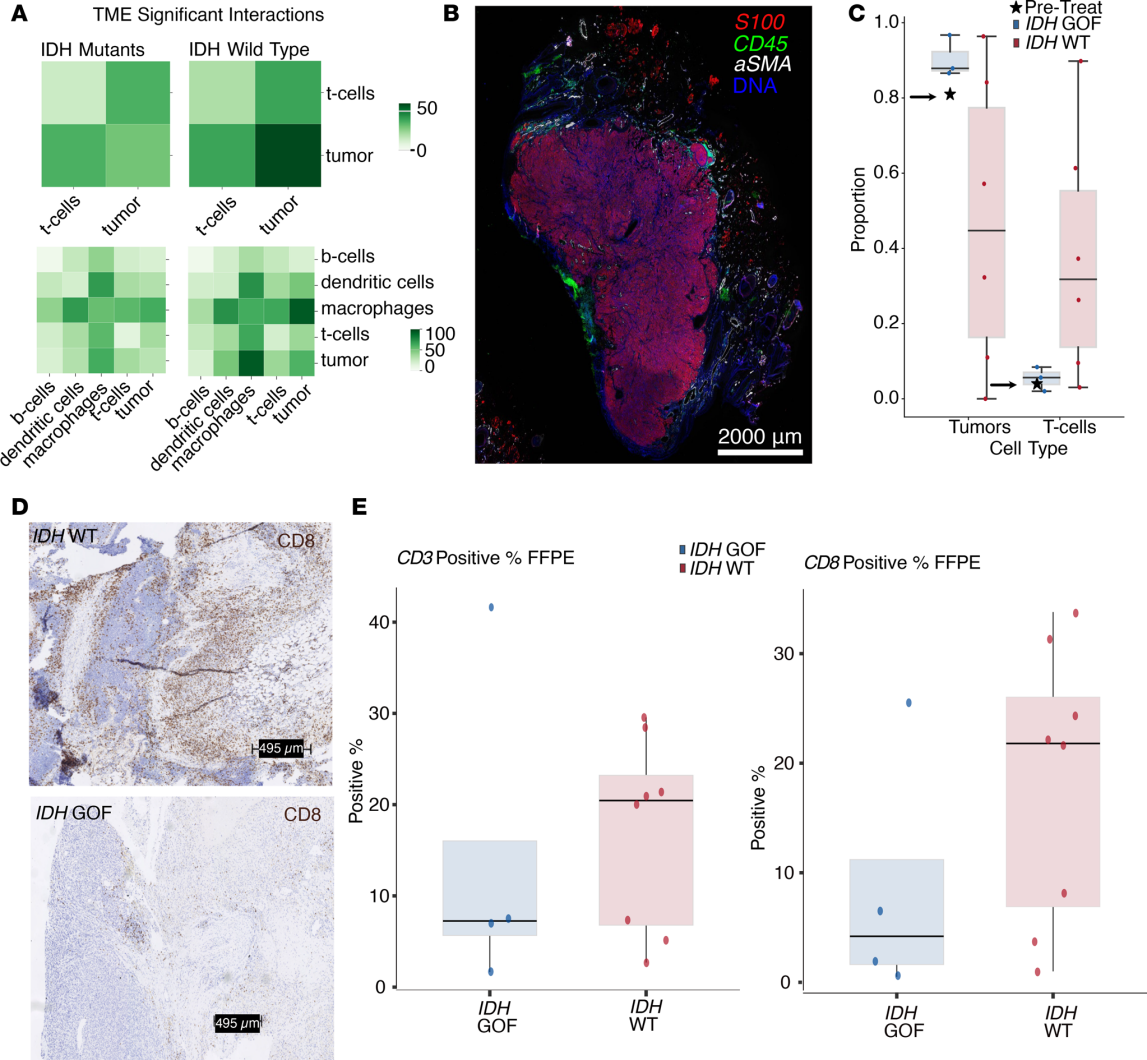
*IDH* GOF mutant tumors have decreased T cell and tumor interactions and a cold tumor environment. (**A**) Heatmap showing the predicted ligand-receptor interactions in each condition. Predicted ligand-receptor interactions (using CellphoneDB) were decreased in mutants, specifically in the T cell/Tumor interactions. (**B**) Like the single-cell data, a pretreatment tumor sample with mutant *IDH* from ref. 14, imaged with t-CyCIF, shows an “immune desert” with few immune cells along the edge of the tumor. Scale bars: 2,000 μm. (**C**) The proportions of tumor cells and T cells in this sample fell within the pattern from single-cell data. Star represents sample shown in **B**. (**D**) Representative staining of *IDH* WT (*n* = 8) and *IDH* GOF (*n* = 4) with CD8 antibody. Scale bars: 495 μm. (**E**) Percentage of total cells identified that were positive for *CD3* or *CD8* split by *IDH* GOF status.

Our analyses of scRNA-seq show that *IDH* GOF tumors include a limited number of T cells, suggesting limited interactions between tumors and immune cells in these tumor types. To explore whether (and how) cells functionally interact within *IDH* GOF and WT tumors, we used CellphoneDB, which allowed us to characterize the number and type of interactions across cell types in *IDH* GOF and *IDH* WT melanomas (13). CellphoneDB uses a curated list of ligand-receptor pairs to analyze whether there is sufficient expression of these pairs within the samples to predict that particular ligand-receptor interaction. Our analysis revealed decreased predicted interactions between tumor cells and immune cells in *IDH* GOF melanomas as compared with the *IDH* WT melanomas (Figure 3A). These results were particularly striking for T cell/tumor interactions (25 in *IDH* GOF vs. 29 in *IDH* WT). In combination with the previous observations, these results led us to hypothesize that *IDH* GOF tumors are characterized by a “cold” noninteractive immune environment.

To directly visualize the tumor microenvironment, we queried published spatial imaging of an *IDH* GOF patient (14). Examining the staining for *S100* (marker for melanoma tumor cells) and *CD45* (marker for leukocytes) revealed broad immune exclusion of the tumor (Figure 3B). This sample displayed a cell type composition consistent with the trend identified from single-cell data (Figure 3C), with a high density of tumor cells observed within the *IDH* GOF tumor. To further explore this conclusion we examined FFPE samples obtained from a complementary cohort of melanoma metastases (Methods). We found that there was a trend of fewer *CD8*^+^ T cells in *IDH* GOF samples (*n* = 4) compared with *IDH* WT samples (*n* = 8), but this was not statistically significant (Wilcoxon’s rank-sum 1-sided, *P* = 0.1414; Welch’s 1-sided *t* test, *P* = 0.1165) (Figure 3, D and E). The same trend was observed using *CD3* as a marker of all T cells (Wilcoxon’s rank-sum 1-sided, *P* = 0.3414; Welch’s *t* test 1-sided, *P* = 0.4057). These trends were in line with the trend identified from single-cell data. These analyses support the conclusion that *IDH* GOF melanomas are characterized by a cold tumor environment.

### Gene expression analysis of IDH GOF melanoma shows global transcriptional silencing.

**Figure 4:**
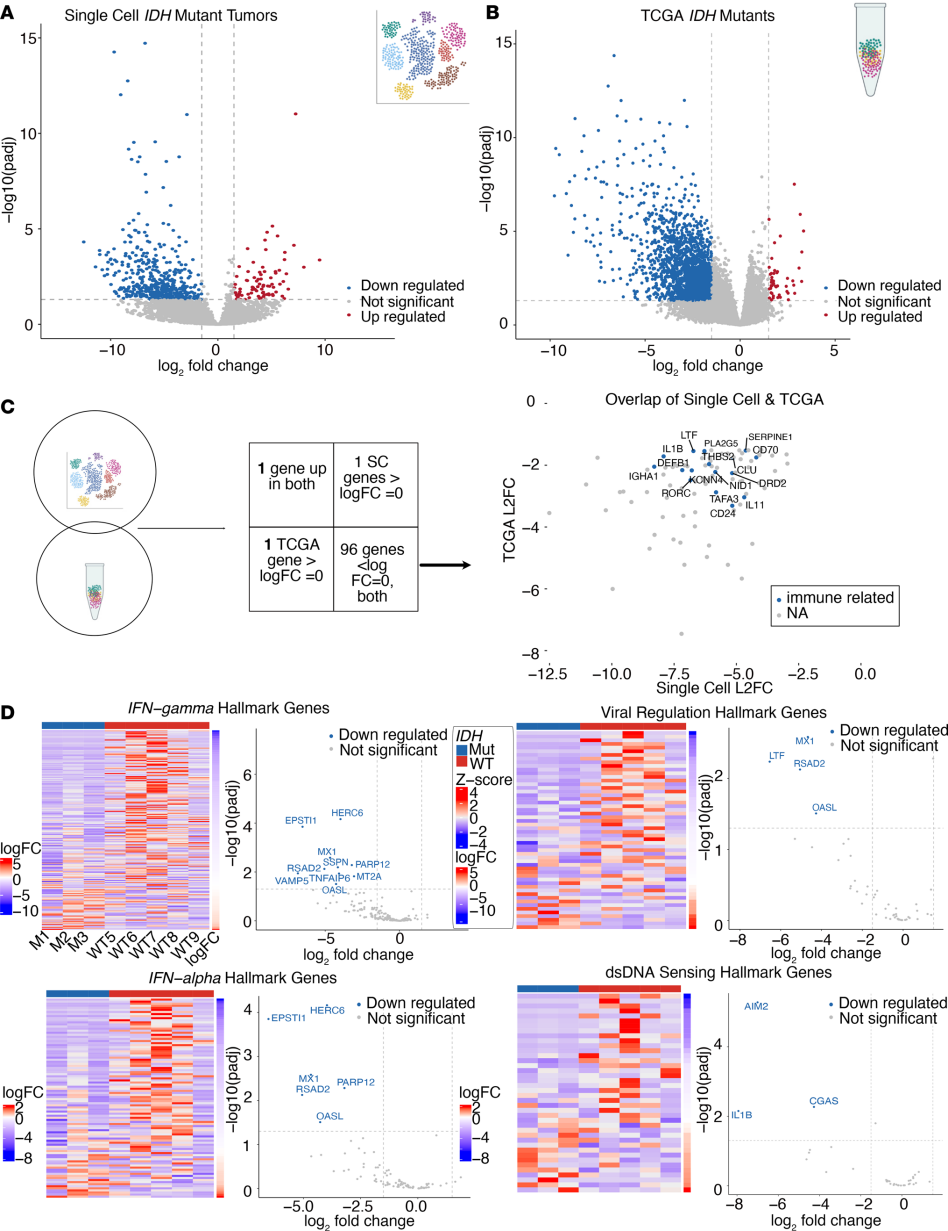
*IDH* GOF melanoma tumors are globally silenced and may promote resistance through dsDNA-mediated viral mimicry. (**A**) Volcano plot of the differentially expressed genes across single-cell melanoma conditions with significantly upregulated genes in red and significantly downregulated genes in blue. All labeled points refer to gene labels. For visualization purposes, 7 genes with log_2_FC < 0 were excluded from the volcano plot (but included in the analysis). Pseudobulk analysis of tumor cells from the single-cell samples demonstrated global gene repression. (**B**) Volcano plot of the differentially expressed genes across TCGA conditions with significantly upregulated genes in red and significantly downregulated genes in blue. For visualization purposes, 8 genes with log_2_FC < 0 were excluded from the plot (but included in the analysis). Bulk RNA-sequencing data from TCGA mirror the global gene repression. (**C**) 96 overlapping significantly differentially expressed genes between the single-cell and TCGA cohort were identified, many of which fell within the immune-related GO terms (blue dots). (**D**) Heatmap showing expression of gene sets with higher expression in red and lower expression in blue. Volcano plots show specific log_2_FC and adjusted P values for each gene in the set between conditions. *IFN*-response Hallmark Genes sets were overall downregulated in *IDH* pseudobulk single-cell data. Omitted for visualization purposes was *MX2* at coordinate (log_2_FC = –8.0522, *P*adj = 1.02029e-19). Viral regulation and cytosolic dsDNA sensing were also downregulated in *IDH* GOF mutants.

Profiling gene expression at single-cell resolution in *IDH* GOF and WT tumors allowed us to isolate the *IDH*-driven changes that are specific to tumor cells. We ran pseudobulk analysis using only the tumor cells from each single-cell sample. Pseudobulk analysis sums all cell profile gene counts from a single sample per gene so that differential gene expression between conditions can be assessed (Methods). One sample (in the *IDH* WT cohort) was excluded from the analysis due to low tumor cell counts (Methods). Comparing *IDH* GOF tumor cells with *IDH* WT tumor cells, we found a global pattern of gene silencing in *IDH* GOF tumor cells, with 586 genes significantly downregulated (log fold change [FC] < –1.5, adjusted *P* value [*P*adj] < 0.05) in *IDH* GOF mutants and only 106 genes upregulated (logFC > 1.5, *P*adj < 0.05) (Figure 4A). A pronounced downregulation of gene expression is consistent with a globally hypermethylated genome in *IDH* GOF tumors, as was previously suggested (5). Interestingly, this global transcriptional regulation was confined to tumor cells and was not seen when we performed a T cell–specific differential gene expression analysis (Supplemental Figure 4).

To understand which groups of genes were being silenced, we performed Gene ontology (GO) term analysis on the downregulated genes identified from the tumor cell pseudobulk analysis. The top 20 enriched biological process GO terms were related to invasive cancer progression such as “angiogenesis,” “ECM organization,” and “immune modulation” (Supplemental Figure 6). We observed that 15 of the top 100 downregulated genes were related to immune regulation, including *CD70*, *IL1B*, and *LTF* (Supplemental Data Values). These results suggest that some of the immune consequences seen in the single-cell data may result from transcriptional changes in the *IDH* GOF melanoma tumor cells.

To validate these results in a larger patient cohort, we analyzed differential gene expression from TCGA skin cutaneous melanoma (SKCM) bulk RNA-seq data (5). TCGA SKCM data include 17 tumors with an *IDH1* or *IDH2* GOF mutation and 431 *IDH* WT samples. We found the same pattern of global silencing, with 2,305 genes downregulated in mutants (logFC < –1.5, *P*adj < 0.05) and 48 genes upregulated (logFC > 1.5, *P*adj < 0.05) (Figure 4B). GO term analysis found immune modulation programs within the most enriched 20 GO terms among downregulated genes (Supplemental Figure 6). These results suggested that the effects of *IDH* GOF mutation on immunologic gene expression programs are consistent across sampling conditions and methods.

To characterize the potential functions of the most consistently dysregulated genes in *IDH* GOF tumor cells, we looked at the overlap of significantly differentially expressed genes between our single-cell and TCGA bulk sequencing data (Figure 4C). This gene set included 99 differentially expressed genes shared across the single-cell tumor cells and TCGA bulk samples. The repressed gene set of 96/99 genes was downregulated in both groups, and 16% of these were related to immune pathways (Figure 4C).

Important genes such as *IL1B*, which has been shown to trigger inflammatory pathways, and *CD70*, which is part of the TNF pathway, are present in this repressed gene set (15, 16). These results together demonstrate that *IDH* GOF melanoma tumor cells are characterized by a highly silenced genome, resulting in downregulation of immune-related transcriptional pathways within the tumor cells themselves.

### Downregulation of double-stranded DNA viral mimicry pathways in IDH GOF mutant melanoma.

To understand how the *IDH* GOF tumor cell transcriptional state results in a melanoma with decreased T cell infiltrate, we examined the function of previously identified innate immune pathways in these cells.

Recently published work in a mouse model of *IDH* GOF cholangiocarcinoma suggested that transcriptional silencing of the *cGAS*-mediated viral mimicry response led to reduced *CD8*^+^ cytotoxic T cell recruitment (17). To see if this same mechanism is present in patients with melanoma, we explored the expression of the double-stranded DNA viral mimicry pathway in our human single-cell tumor cell data, comparing the expression of “*IFN-Alpha* Response Hallmark Gene Set” and “*IFN-Gamma* Response Hallmark Gene Set” (Figure 4D) (18). We saw that almost all genes in these sets were less expressed in *IDH* GOF melanoma (log_2_FC < 0) and all significantly differentially expressed genes were downregulated in *IDH* GOF melanoma (*P*adj < 0.05, l2FC < –1.5). For *IFN-Alpha*, 72/96 genes had log_2_FC <0 and 6 of those genes had *P*adj <0.05 and l2FC <–1.5. For *IFN-Gamma*, 150/196 genes had log_2_FC <0 and 11 of those genes had *P*adj <0.05 and l2FC <–1.5. This result suggests that downregulation of *IFN* signaling in *IDH* GOF melanoma cells may lead to decreased recruitment of T cells. The *IFN* response has been previously linked to the viral mimicry pathway (19). We next examined the expression of the “Viral Regulation Hallmark Gene Set” and the “Cytosolic dsDNA Sensing KEGG Gene Set” (18). For viral regulation, 40/54 genes had log_2_FC <0 and 4 of those genes had *P*adj <0.05 and l2FC <–1.5. For cytosolic dsDNA sensing, 26/57 genes had log_2_FC <0 and 3 of those genes had *P*adj <0.05 and l2FC <–1.5. This analysis showed that the majority of genes in these gene sets are less expressed in *IDH* GOF melanoma, and all significantly differentially expressed genes were downregulated (Figure 4D). These results suggest that the downregulation of dsDNA viral mimicry pathways, previously suggested in mouse models of *IDH* GOF cholangiocarcinoma, are relevant in human melanoma and provide a mechanism by which *IDH* GOF mutant melanoma may evade immune infiltration.

### Transcriptional repression is associated with DNA methylation in IDH GOF melanoma.

**Figure 5:**
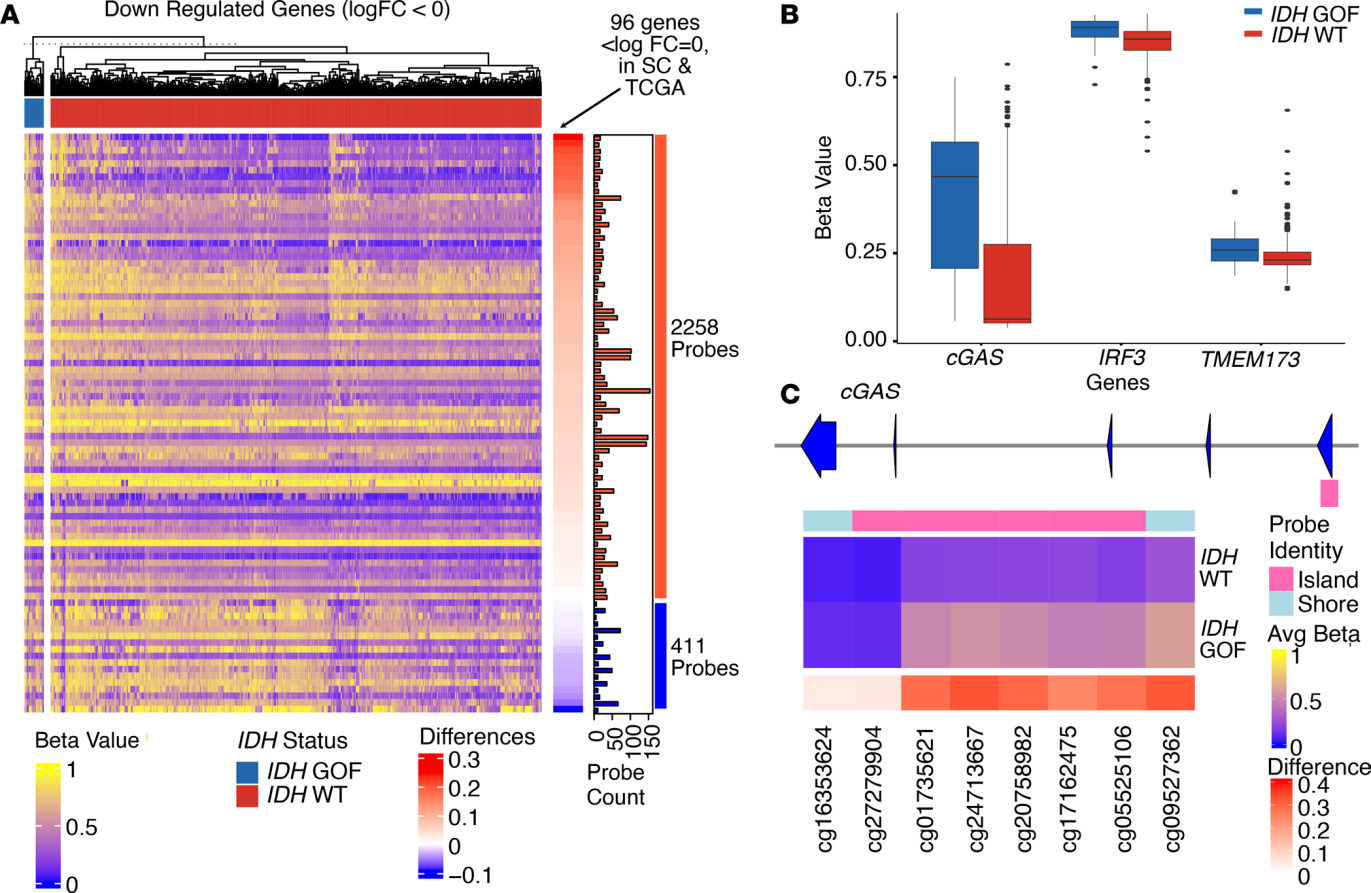
*IDH* GOF melanoma demonstrates global methylation and silencing of the *cGAS* pathway. (**A**) Heatmap of 96 repressed gene set methylation probes from TCGA averaged by gene and split by mutational status, with yellow representing more methylated and blue representing less methylated. Average difference between β values by category shown to the right, with red representing more methylated in *IDH* GOF and blue representing less methylated in *IDH* GOF. Methylation probes associated with the 96-gene signature obtained from single-cell and TCGA data (from Figure 4C) reveals that the majority are more methylated in *IDH* GOF samples than WT samples. (**B**) Box plot showing the averaged β values per gene split between conditions. (**C**) Testing components of the viral mimicry *cGAS* pathway shows higher methylation in *IDH* GOF samples, especially for *cGAS*. Heatmap of probes associated with the *cGAS* gene, with yellow representing most methylated and yellow representing less methylated. The differential *cGAS* methylation is enriched in CpG islands (in pink).

To understand if high levels of DNA methylation effect gene expression in *IDH* GOF melanoma, we analyzed the methylation status of the probes associated with the 96 genes in the repressed gene set in both single-cell and TCGA RNA-seq data (Figure 4C). Using TCGA 450K Illumina array, we calculated the differential methylation between *IDH* GOF and *IDH* WT melanoma by comparing average β values (a measure of methylation proportion of a CpG probe) at each of these genes (Methods). The majority of the repressed gene set’s genes, 67/96 genes, were more methylated in the *IDH* GOF melanomas compared with *IDH* WT tumors, suggesting that increased methylation is associated with gene silencing (Figure 5A). We then examined the methylation status of downregulated *cGAS* pathway gene-associated probes. This analysis demonstrated that *cGAS*, *IRF3*, and *STING* were on average more methylated in *IDH* GOF samples compared with *IDH* WT samples (Figure 5B). Of these, the probes associated with the *cGAS* gene demonstrated the greatest difference in average β value methylation between *IDH* GOF and *IDH* WT. We then queried the location within the gene of these probes and found that the hypermethylation at *cGAS* was particularly evident at CpG island probes, suggesting functional silencing due to disruption of transcriptional initiation (Figure 5C) (20).

## Discussion

*IDH* GOF mutations are present in approximately 5% of melanomas and result in DNA hypermethylation. Understanding the effect of this genomic alteration on tumor and clinical phenotypes will not only provide insight into the treatment of patients with *IDH* GOF mutant melanoma but will also inform our understanding of the 20% of melanomas that are DNA hypermethylated. Here, we compared the clinical phenotype and tumor microenvironment of *IDH* GOF mutant tumors (*n* = 19 clinical patients, *n* = 3 single-cell tumor samples, *n* = 4 FFPE tumors) and matched *IDH* WT melanomas (*n* = 70 clinical patients, *n* = 6 single-cell tumor samples, *n* = 8 FFPE tumors). Both scRNA-seq and spatial analyses found that the tumor microenvironment of *IDH* GOF melanoma is characterized by a decreased immune infiltrate. This finding was supported by similar trends observed in a complementary cohort analyzed by IHC. The GEPs of the few T cells present in *IDH* GOF melanomas indicated that the T cell states were more immunosuppressive, with fewer interactions with tumor cells. In line with these differences observed in the immune compartment, we found that patients with *IDH* GOF mutant melanoma responded relatively poorly to single-agent anti-*PD1* therapy in the metastatic setting (10).

Previous work showed that *IDH* GOF mutation in epithelial tumor cells results in the suppression of the cell-intrinsic *cGAS* dsDNA sensing pathway, leading to decreased *IFN* production by these tumor cells and therefore poor recruitment of TILs (17). We observe a similar downregulation of the *cGAS* pathway in *IDH* GOF melanoma (among many other downregulated genes), which might explain the low tumor infiltration and response to immunotherapy. Inhibition of the *IDH* GOF protein by the FDA-approved drug ivosidenib may reverse this suppression and restore immune function. Recent work in *IDH* GOF in cholangiocarcinoma found that a combination of *IDH* GOF inhibitor and anti-*CTLA* therapy led to an antitumor response in mouse models (21). By blocking the tumor-intrinsic effects of *IDH* GOF the authors restored recruitment of *CD8*^+^ T cells in mouse models of cholangiocarcinoma. Our data from human melanoma samples suggest that this treatment approach, or alternative combination therapies, may improve outcomes in patients with *IDH* GOF melanoma. To date, however, there are no reported clinical trials exploring the use of ivosidenib in *IDH* mutant metastatic melanoma.

Intralesional therapies capable of stimulating the cold immune environment and increasing TILs may represent an approach to inducing immune-responsiveness in *IDH* GOF melanoma. Interestingly we note 3 patients with *IDH* GOF mutant melanoma at our institution who received TVEC for treatment of metastatic disease (Supplemental Figure 7). Two of these patients had complete responses to this therapy, and both remained disease free for over a year. The recognition that patients with *IDH* GOF mutant melanoma may harbor intrinsic resistance to anti-PD1 therapy should encourage rapid identification of resistance and tailored therapeutic approaches.

Temozolomide (TMZ) is an alkylating agent that is used in brain tumors, and *IDH* GOF in gliomas has been shown to be a positive predictive biomarker for response to TMZ. This would predict that *IDH* GOF melanomas may be more sensitive to TMZ as well. In melanoma TMZ is currently used only in the palliative setting for patients with treatment-resistant brain metastases. In a phase II study of TMZ in advanced melanoma performed prior to the introduction of immunotherapy, 3 of 56 patients treated with TMZ showed a complete response (22, 23). It is possible that these exceptional responders had an *IDH* mutation, but this trial was reported in 1995, long before NGS was used to help guide melanoma treatment. The proposed mechanism of action of TMZ in *IDH* GOF tumor cells is to induce DNA damage and promote apoptosis. It is reasonable to consider testing combination therapies including TMZ in preclinical models of *IDH* GOF melanoma. Along these lines, we speculate that epigenetic agents such as DNA demethylating drugs may provide a potential benefit to the subset of patients with melanoma with DNA hypermethylation. *IDH* GOF melanoma tumors may provide mechanistic insights into hypermethylated melanomas, which represent approximately 20% of all melanomas in TCGA cohort (5). The most highly methylated melanomas within TCGA cohort were suggested to have a relatively poor prognosis. Future studies to understand how the mechanisms of immune evasion identified in *IDH* GOF melanoma may apply more broadly to hypermethylated melanomas will yield insight into immunotherapy resistance and treatment approaches relevant to all patients with melanoma.

## Methods

### Sex as a biological variable

Patients with melanoma were included in both retrospective chart review and sample collection regardless of sex, and no biological significance was found.

### Survival analysis

Patients with *IDH* GOF mutant cutaneous melanoma were identified using an institutional genetic testing tool with date of diagnosis between 2010 and 2023 (8). Only *IDH* GOF patients that received first line anti-*PD1* therapy in the metastatic setting were included in analysis (*n* = 19). A cohort of *IDH* WT patients that underwent genetic testing during the same time span were identified as acting as controls. We matched these cohorts on sex, age, primary location, and primary histopathologic features (Supplemental Figure 10). Overall survival was calculated for each patient as the period between the start of their first line of anti-*PD1* therapy in the metastatic setting and the date of death or last follow-up appointment.

Recurrence-free survival was calculated as the time period between the start of anti-*PD1* treatment and the first new tumor growth measured by radiographic detection or biopsy. Response was classified using the best response to the line of treatment using the clinician’s judgment following the RECIST criteria (24). Patients classified as responders were those patients with complete or partial response to treatment. Nonresponders were defined as patients whose best response was progressive or stable disease.

Presence of significant differences in demographic and histopathologic features was assessed using a Fisher’s exact test to check in R (version 2023.06.2+561) (25). Kaplan-Meier survival analysis was performed using the survival package (version 3.5) and survminer package (version 0.4.9), testing for overall survival and progression-free survival as defined above (26, 27). A log-rank test was used to compare overall survival and progression-free survival (test results were identified as significant if *P* < 0.05). Kaplan-Meier survival analysis, swimmers plots, and bar plots of survival results were visualized using ggplot2 (version 3.5.0) (28). A Fisher’s exact test was used to compare the distribution of responders versus nonreponders in *IDH* GOF versus *IDH* WT patients and identify significant differences.

### Gene expression analysis of TCGA

The Cancer Genome Atlas Skin Cutaneous Melanoma Genomic Data Commons gene expression data from bulk RNA-seq data in the format STAR counts was downloaded using TCGAbiolinks (version 2.30.4) (29). Differential gene expression analysis was performed in R (version 2023.06.2+561) using DeSeq2 (version 1.42.1), comparing *IDH* GOF mutants (samples with *IDH1* GOF R132 and *IDH2* GOF mutations) (*n* = 17) versus WT (*n* = 431) patients (30).

Patients with unknown significance *IDH* mutations (i.e., not R132) or noncutaneous origin (as defined in the multinomic_melanoma_study_2019 TCGA patient characteristics file from ref. 31, downloaded from Github; https://github.com/ianwatsonlab/multiomic_melanoma_study_2019/blob/master/data/dat_clin.tsv) were excluded from analysis. Genes not expressed (<5 raw counts) in any sample regardless of *IDH* status were not considered in the analysis. Genes were considered differentially expressed following these criteria: *P*adj < 0.05 and log FC >|1.5|.

GO term analysis was performed using ClusterProfiler (version 4.10.1) (32), using as background all genes expressed >5 raw counts across all samples. Purity of samples was estimated using published ESTIMATE scores, downloaded from Github (https://github.com/ianwatsonlab/multiomic_melanoma_study_2019 from ref. 31).

### scRNA-seq and analysis

#### Library preparation.

scRNA-seq was performed on *IDH* GOF (*n* = 3) and *IDH* WT (*n* = 6) samples, matched on treatment setting, sample location, primary type, stage at time of sample, age, and sex, in that preferential order. Samples were collected between 2020 and 2023. The tumor samples were collected surgically from patients with metastatic melanoma under protocol 11-181 at Massachusetts General Hospital. Freshly resected tumor biopsies were processed using the Human Tumor Dissociation Kit (Miltenyi Biotec, 130-095-929) according to established protocols (33, 34). Biopsies were minced in 1.5 mL Eppendorf tubes containing RPMI medium and the kit-provided enzyme mix (H, R, A) and then incubated at 37°C in a thermomixer with continuous shaking at 400 rpm for 15 minutes. Following enzymatic digestion, cell suspensions were filtered through a 50 μm filter (Sysmex, 04-004-2327) and washed with 5 mL RPMI supplemented with 10% heat-inactivated FCS. The filtrate was centrifuged at 252 RCF for 5 minutes at 4°C, the supernatant was discarded, and the pellet was treated with ACK lysis buffer (Gibco, A1049201) to remove red blood cells. After centrifugation under the same conditions, cells were resuspended in RPMI with 10% heat-inactivated FCS. Cell concentration and viability were assessed using trypan blue exclusion and a Bright-Line hemocytometer (Hausser Scientific, catalog HS-1492). For samples with viability <70%, viable cells were enriched using a modified EasySep Dead Cell Removal (Annexin V) protocol (STEMCELL, 17899), as described by Fang (34). Post-enrichment, viability, and counts were reassessed before scRNA-seq preparation.

Gene expression libraries were generated using 10x Genomics Chromium NextGEM Single Cell V(D)J Reagents Kits v1.1 (PN-1000165, PN-1000120, PN-1000213) and Single Cell 5′ Reagent Kits v2 (PN-1000263, PN-1000286, PN-1000215), following the manufacturer’s instructions. Quality control of amplified cDNA and final libraries was performed using the Qubit dsDNA High Sensitivity Kit (Invitrogen, Q32854) and Agilent High Sensitivity Bioanalyzer DNA Kit (5067-4626).

Sequencing was conducted on an Illumina NextSeq 500 using the 75-cycle kit (20024906). Read configurations were as follows: NextGEM v1.1: read 1, 26 cycles; read 2, 55 cycles and NextGEM v2: read 1, 26 cycles; read 2, 46 cycle.

#### Data processing and quality control.

Cells by gene matrices were generated using Cellranger-7.0.0 aligned to reference transcriptomGRCh38-2020-A. Ambient RNA was removed using Cellbender run on each sample (version 0.3.0) (35) with epochs = 150, expected cell number from each sample’s Cellranger run, and learning rate = 1e-4.

Analysis was performed on all samples using Python (version 3.11) through Scanpy (version 1.9.6), Anndata (version 0.10.4), and Pandas (version 1.4.4) (36–38).

Cells with cytosolic debris were identified using expression of *MALAT1* (39, 40). The distribution of *MALAT1* expression was plotted for each sample, and cells below the local minima were excluded from further analysis (Supplemental Figure 7). Cells with fewer than 200 genes and genes present in fewer than 3 cells in the merged matrix were removed from further analysis. In each sample, cells with high mitochondrial contents were eliminated (thresholds shown in Supplemental Figure 3).

MAD filtering was run to remove remaining outliers for selected samples that had outliers (Supplemental Figure 7). Scrublet was used to detect and remove doublets; doublets were defined in each sample matrix as barcodes/cells with a doublet score ≥0.2 (version 0.2.2) (41). Highly variable genes were calculated in Scanpy with min_mean = 0.0125, max_mean = 3, min_disp = 0.5, resulting in 2,867 genes.

Neighbors were calculated using 40 PC and then used for UMAP calculation.

#### Cell type identification.

Samples were then clustered using the Leiden algorithm (UMAP version 0.5.5, leidenalg version 0.10.1, resolution = 1). Clusters of immune, epithelial, and tumor cells were identified using canonical marker gene lists and infercnvpy (version 0.4.4) (42–44).

The top 15 differentially expressed genes for each cluster were manually compared with marker genes taken from published sources (42, 45) to label cell types.

#### Cell type composition.

The proportion of each cell type count divided by total cell count per sample was calculated for each sample (Pandas, version 1.4.4) (38). scCODA was used to test differential cell composition using fibroblasts as a baseline, as predicted by the algorithm (version 0.1.9) (9). Proportions were analyzed by Beysian fit (Figure 2B) Results were visualized using seaborn (version 0.13.1) and matplotlib (version 3.8.2) (46, 47).

#### T cell states analysis.

T cells were identified using the classification cluster of the overall UMAP and expression of CD3E. These cells from all samples were integrated using Harmonypy, defining each sample as a batch (version 0.0.9) (48). We began by looking at known T cell subtype markers to identify clusters (43). We identified T cell states using Hotspotsc (version 1.1.1) and found an optimal number of 9 modules that broadly corresponded to T cell states (11). Using min_gene_threshold = 45, the local correlation plot demonstrated that genes meaningfully split cells without dividing functional groups. The identity of these modules was identified using the ontologies of the top 20 genes associated with each module. For modules 7 and 8, the genes present were not clearly indicative of a functional T cell state but rather of activation more generally. Therefore, these modules were not used to classify a functional state.

These modules were also lowly used and diffused across T cells, rather than representing a distinct subidentity. Cells were then classified as belonging to a particular cell state by assigning them to their respective top module. For those cells that had module 7 or module 8 as their top state, their next highest module was used.

The proportion of each T cell subtype count divided by total T cells from that sample was calculated. These were grouped by condition and reported as a box plot (Figure 2). These classifications were supported by gene set scoring using the scanpy.tl.score_genes function, using bins = 25 in Scanpy (version 1.9.6) (37, 43). These classifications showed the same trends as Hotspot (Supplemental Figure 3).

Pseudobulk testing was performed on the tumor cells from each single-cell sample (i.e., the GEPs of all tumor cells within a sample were summed per sample to generate one pseudobulk mRNA profile). One *IDH* WT sample (WT4) was excluded as it had a particularly low tumor cell count (50 cells). Counts were normalized per sample. DeSeq2 and GO testing were then run under the same conditions as the bulk RNA data (30,32).

CellphoneDB was used to assess predicted ligand-receptor interactions (version 5.0.0) (13). Samples were grouped by condition (*IDH* GOF vs. *IDH* WT). The gene expression analysis was then run with the identified cell type for the grouped conditions. Predicted significant interactions (*P* < 0.05) were compared between conditions to understand the differences of the predicted activity in the tumor microenvironment.

### FFPE samples

Samples were identified from the Massachusetts General Hospital institutional biobank. Samples from metastatic sites of *IDH* GOF untreated tumors (*n* = 4) were matched to samples from metastatic sites from *IDH* WT tumors (*n* = 8) based on site, stage at time of collection, and comutations in that order. Of these samples, 1 sample overlapped with our scRNA-seq cohort (Supplemental Figure 9). These samples were then stained for *CD3* (Dako, A0452, 1:250) and *CD8* (Dako, M7103, 1:100) to visualize the immune infiltrate. A dermatopathologist manually highlighted regions that were considered to be tumors to see the TILs.

QuPath software was then used to call cells within these regions using the “Positive Cell Detection” feature with default cell size settings and identify the cell’s positivity. The percentage of positive cells was calculated as positive cells/total cells identified. Statistical analysis was performed using a nonparametric Wilcoxon’s rank-sum using 1-tailed Welch’s *t* test, GOF vs. WT, alternative = “less” from the stats package in R (version 4.6.0).

### Overlapping comparisons

Significantly differentially expressed genes (*P*adj < 0.05) between TCGA and single-cell data were compared. A total of 99 genes were found to be in common between the groups. The downregulated genes (logFC < 0) were compared to see what was common between sampling conditions. Genes were classified by their belonging to 1 of the top 30 GO terms found for single-cell and TCGA GO testing.

### Methylation data analysis

Methylation β values 450K Illumina array were downloaded from TCGA SKCM GDC data set using “TCGAbiolinks” (version 2.30.4) (29). β Values were averaged across each condition, i.e., *IDH* GOF (*n* = 17) and *IDH* WT (*n* = 431). Heatmaps demonstrating key probes associated with *cGAS-STING* pathway genes were created using ComplexHeatmaps (version 2.18.0) (49). Probes’ association with each gene was determined using the Illumina 450K array mapping files (downloaded from https://support.illumina.com/downloads/infinium_humanmethylation450_product_files.html).

### Statistics

*P* values of less than 0.05 were considered significant for all statistical testing. Testing was performed for each data point as described in the text and Methods.

### Study approval

Clinical data from patients was collected under protocol 23-692, which was approved by the Dana-Farber Cancer Institute IRB. Written informed consent for tissue collection was obtained from patients prior to tissue collection under protocol 11-181, which was approved by the Dana-Farber Cancer Institute IRB.

### Data availability

All calculated values from analysis are available in the Supporting Data Values file. Processed scRNA-seq data are available at https://singlecell.broadinstitute.org/single_cell/study/SCP3135 Raw sequencing data are available in NCBI BioProject under accession PRJNA1435432. Supporting data will be available in the Supporting Data Values file.

## Author contributions

SC: resources, conceptualization, supervision, formal analysis, investigation, visualization, and writing. ETT: conceptualization, supervision, formal analysis, investigation, visualization, and writing. ES: formal analysis, investigation, data curation, visualization, and writing. LP: investigation. MJW: review of the manuscript. RWJ: resources and investigation. DNE: data curation. NB: review of the manuscript. BB: conceptualization and review of the manuscript. MSF: resources, investigation, and review of the manuscript. CL: data analysis. GMB: resources, supervision, and review and editing of the manuscript.

## Conflict of interest

SC has sponsored research agreements through her institution with Astellas and AstraZeneca. GMB has sponsored research agreements through her institution with Olink Proteomics, Teiko Bio, InterVenn Biosciences, Palleon Pharmaceuticals, Astellas, and AstraZeneca. She served on advisory boards for Iovance, Merck, Moderna, Nektar Therapeutics, Novartis, Replimune, and Ankyra Therapeutics. She consults for Merck, InterVenn Biosciences, Iovance, and Ankyra Therapeutics. She holds equity in Ankyra Therapeutics. RWJ is a member of the advisory board for and has a financial interest in Xsphera Biosciences Inc., a company focused on using ex vivo profiling technology to deliver functional, precision immune-oncology solutions for patients, providers, and drug development companies. RWJ has received honoraria from Incyte (invited speaker), G1 Therapeutics (advisory board), and Bioxcel Therapeutics (invited speaker). RWJ has an ownership interest in US patents US20200399573A9 and US20210363595A1. RWJ’s interests were reviewed and are managed by Massachusetts General Hospital and Mass General Brigham in accordance with their conflict-of-interest policies. BB declares outside interests in HiFiBio, Arsenal Biosciences, Cell Signaling Technologies, and Chroma Medicine.

## Funding support

This work is the result of NIH funding, in whole or in part, and is subject to the NIH Public Access Policy. Through acceptance of this federal funding, the NIH has been given a right to make the work publicly available in PubMed Central.

- KL2TR002542 award through Harvard Catalyst | The Harvard Clinical and Translational Science Center (National Center for Advancing Translational Sciences, NIH) to SC.
- Adelson Medical Research Foundation Grant to SC, GMB, and MSF.
- School of Biological and Behavioural Sciences (Faculty of Science & Engineering) at Queen Mary University of London to ETT.
- Seriver Laboratories to NB.
- Massachusetts Life Sciences Center Research Infrastructure Program in support of the Mass General Cancer Center Tumor Cartography Center to RWJ.

## Supplementary Material

Supplemental data

Supporting data values

## Figures and Tables

**Table 1:**
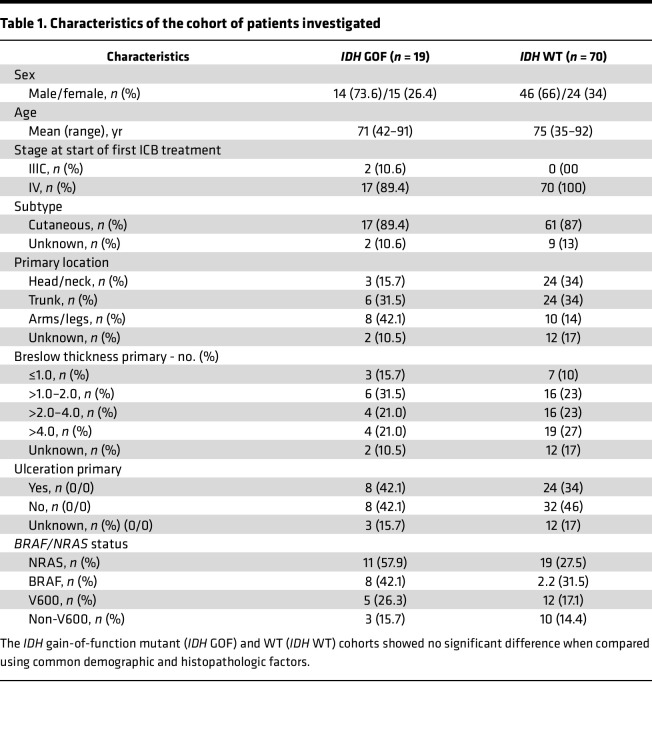
Characteristics of the cohort of patients investigated

## Version 1 04/09/2026

In-Press Preview

## Version 2 06/08/2026

Electronic publication

## Version 3 07/02/2026

Authorship note added
