# Supplementary material for: Loss of tumor-infiltrating lymphocytes and poor response to immunotherapy in IDH GOF mutant melanoma
Source: JCI Insight. 2026 Apr 9;11(11):e195384. doi: 10.1172/jci.insight.195384 (PMC13313554; doi:10.1172/jci.insight.195384)
Supplement: Supplemental data [file jciinsight-11-195384-s007.pdf]

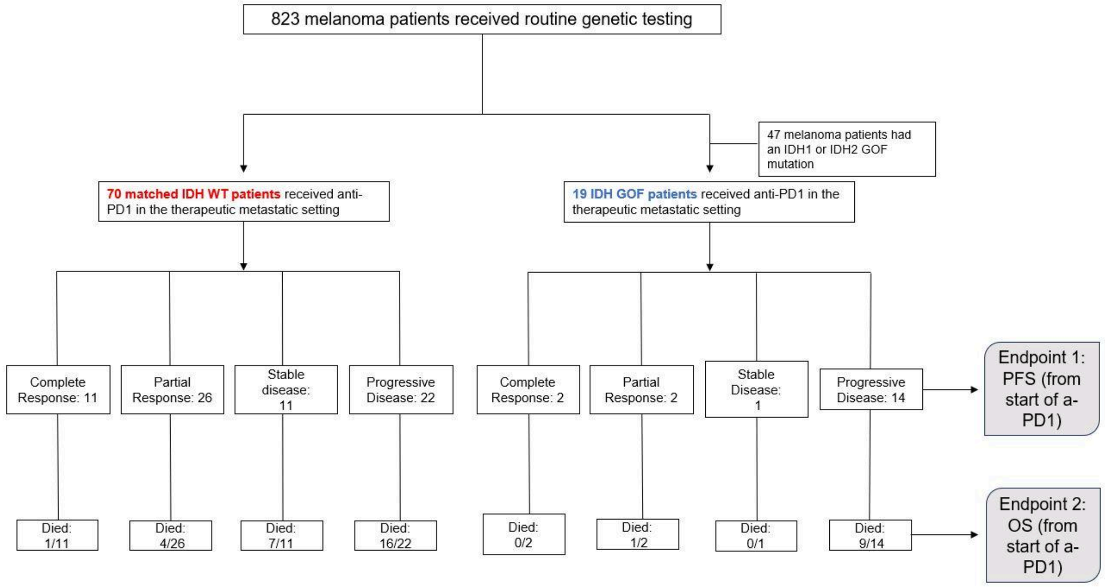

Supplementary Figure 1: Selection and measurement criteria of anti-PD1 cohort for IDH mutant and wild type cohorts measured PFS and OS.

Characteristic - n (%)	IDH Mutants (n=3)	IDH WT (n=6)
**Age Average (range)**	76	63.0
Sex		
male	2 (66.7)	4 (66.7)
female	1 (33.3)	2 (33.3)
**Treatment Setting**		
pre-tx	3 (100)	6 (100)
**Sample Location**		
lymph node	1 (33.3)	2 (33.3)
soft tissue	2 (66.6)	4 (66.7)
**Primary Type**		
Cutaneous	2 (66.7)	4 (66.7)
Unknown Primary	1 (33.3)	2 (33.3)
**Stage at time of sample**		
IIIb/C	1 (33.3)	2 (33.3)
IV (M1a)	2 (66.6)	4 (66.7)

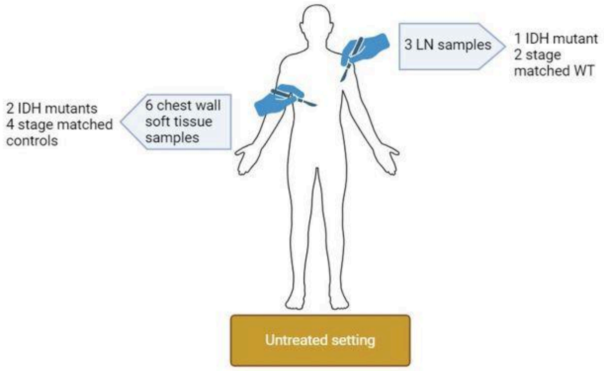

Supplementary Figure 2. Testing of common confounding factors in single cell data between IDH mutant and wild type sample cohorts resulted in well matched cohorts.

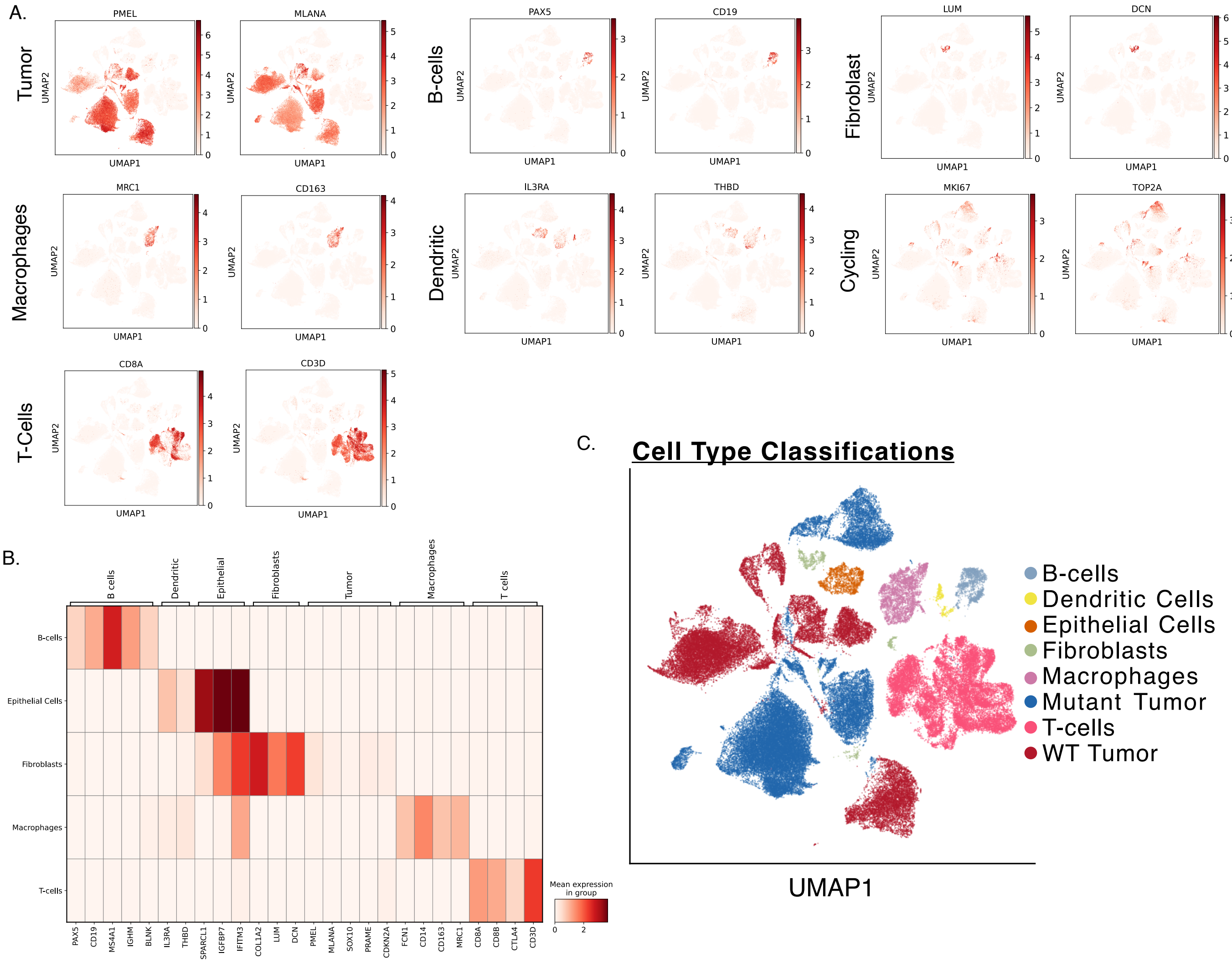

Supplementary Figure 3. Identification of Cell Types Through Gene Expression. Common marker genes demonstrate cluster identities (A). These classifications are supported by both top 15 differentially expressed genes per cluster as well as heat map of markers (B,C)

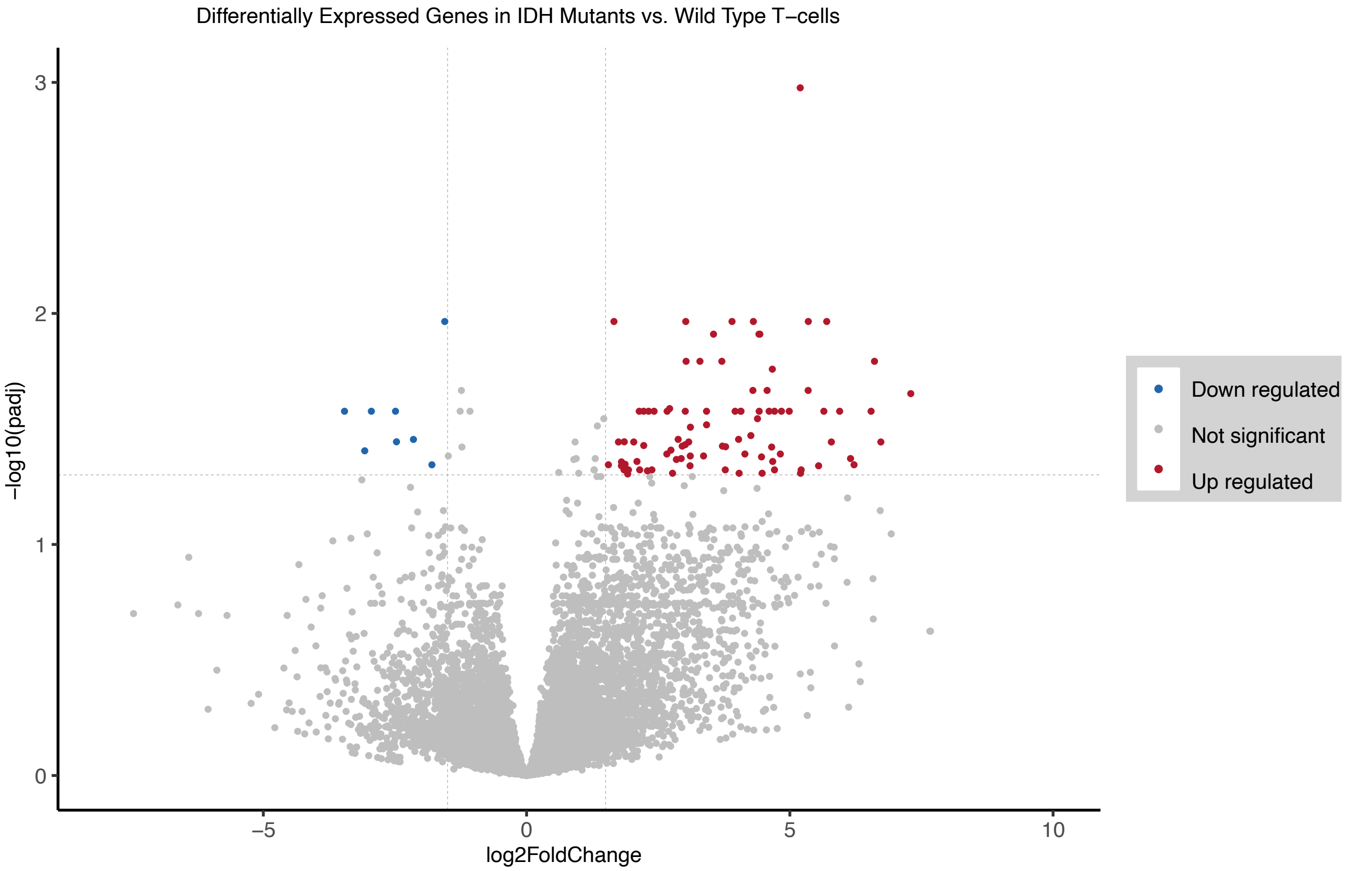

Supplementary Figure 5: pseudo-bulk differential gene expression analysis of T-cells in IDH GOF vs. WT. Differential gene expression shows no large shift indicative of silencing.

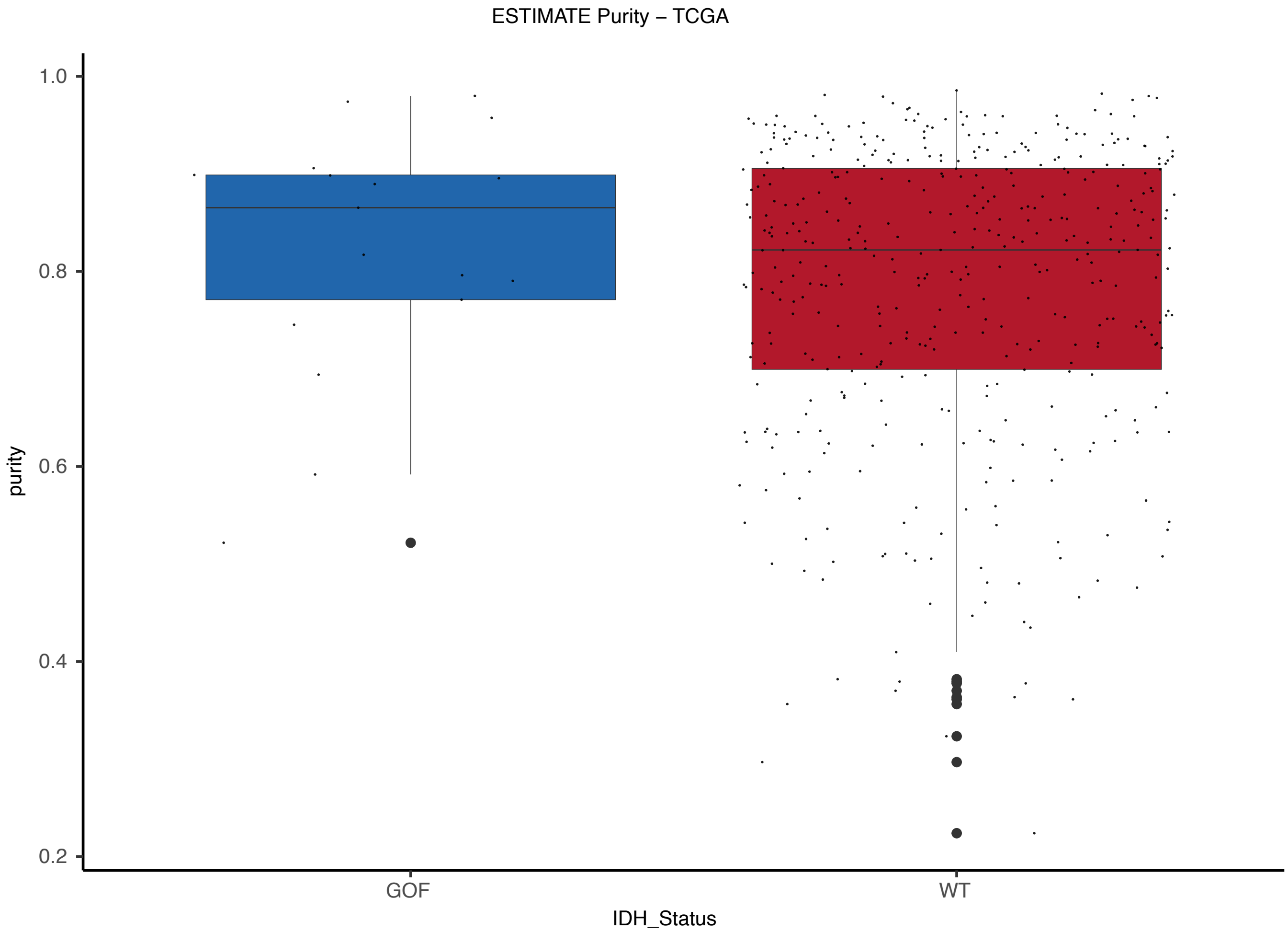

Supplementary Figure 6: Purity ofTCGA calculated between IDH GOF mutants and wild type patients. IDH GOF have higher on average purity.

A.

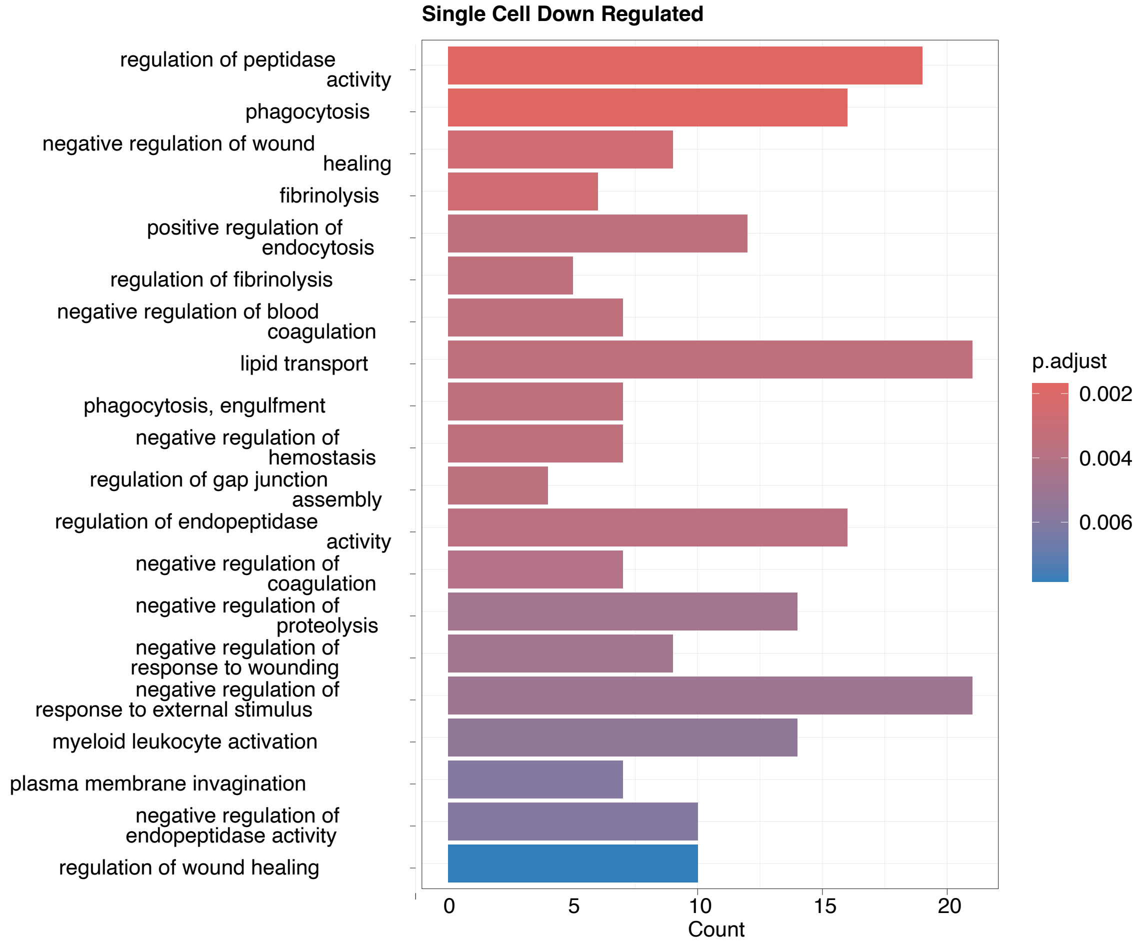

B.

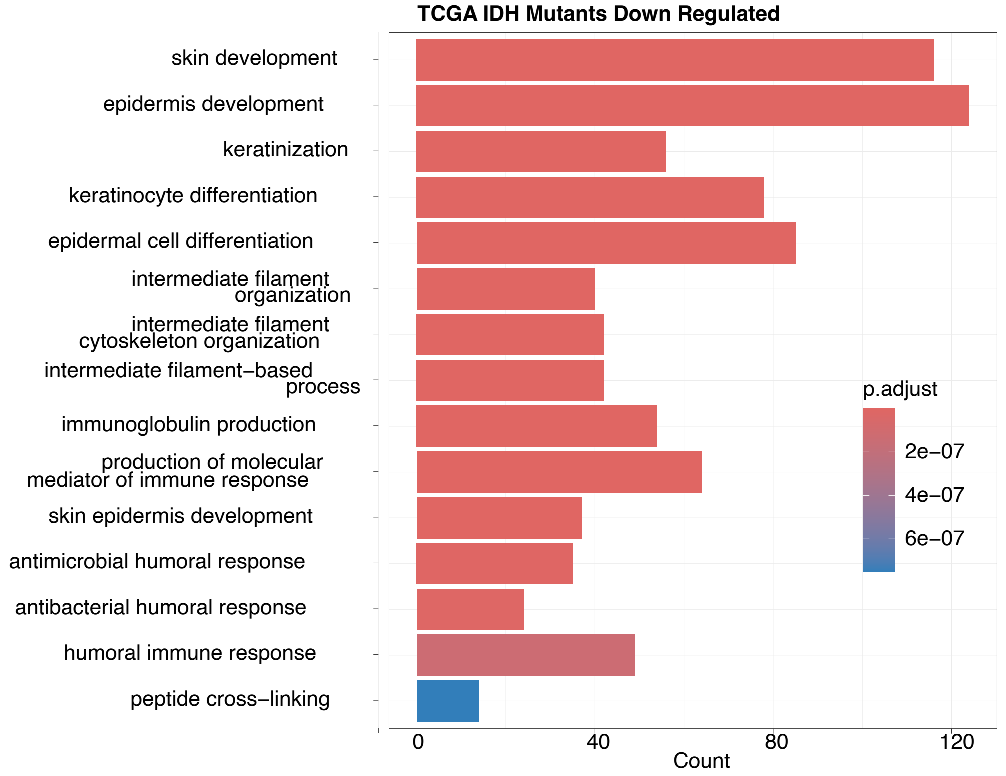

Supplementary Figure 7: GO Term analysis on the down-regulated genes in IDH GOF revealed the metastatic potential of the tumor cells (A). There were immune modifications in the down-regulated terms from the TCGA (B).

Sample	IDH Status	Estimated Initial # of Cells	Thresholding MAD	MLAT local minima	HQ cell count	Scrublet Threshold
M123	Mutant	7,097	no	-1.3	6,566	0.2
M329	Mutant	22215	no	-3.5	20786	0.2
M301	Mutant	6996	no	-1.4	5724	0.2
M200	Wild Type	7852	yes	-4	6494	0.2
M196	Wild Type	7976	yes	-2	5999	0.2
M264	Wild Type	11651	no	-2	10641	0.2
M279	Wild Type	5707	no	-1.4	4129	0.2
M234	Wild Type	9395	yes	-0.7	4879	0.2
M343	Wild Type	8866	no	-3	6708	0.2
**Totals**		87,755			71,926	

Supplement Figure 8 Single Cell RNA Sequencing Quality Control Metrics.

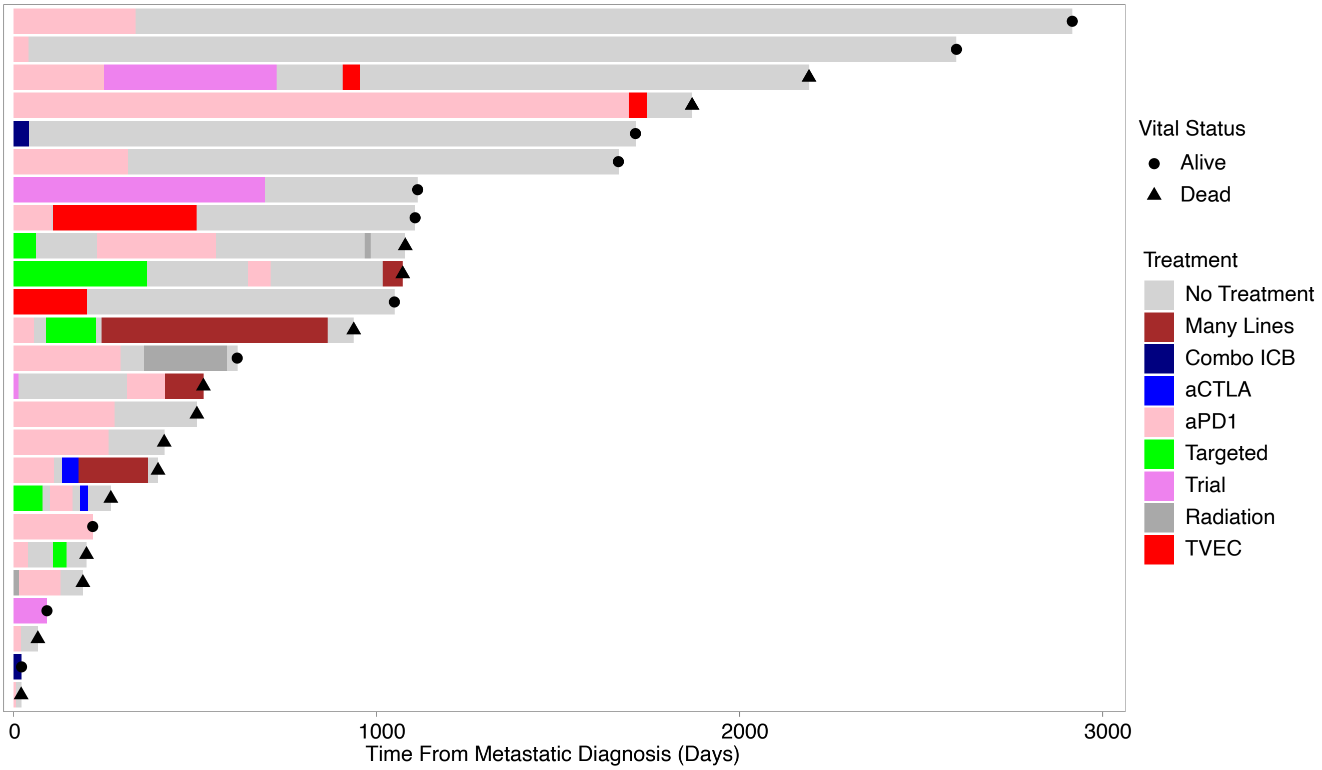

Supplement Figure 9 Swimmers Plot demonstrates the metastatic setting treatment course of IDH GOF mutant patients. Many lines of treatment was defined as >3 in a period of 3 months.

IDH Status	Site	Mutations	Positive % CD3	Positive % CD8	% Immune Cells In Single Cell Cohort
GOF	LN	NRAS, IDH1	1.65	0.5	n/a
GOF	LN	NRAS, IDH1	41.6	25.6	n/a
GOF	LN	SRC, CDKN2A, TERT, IDH1, BRAF	7	6.4	n/a
GOF	LN	FBXW7, IDH1, ATM, NF2, TERT and BRAF V600	7.5	2	8.4
WT	LN	NRAS, TERT, DDX3X, and RNF43	20.9	24.3	n/a
WT	LN	MAP3K1 and TERT	21.45	22	n/a
WT	LN	BRAF	20	21.6	n/a
WT	LN	SMARCA4, TERT, DDX3X, BRAF and TP53	28.5	31.4	n/a
WT	LN	DAXX, CDKN2A, TERT and NRAS.	7.325	3.6	n/a
WT	LN	NRAS, TERT, CDKN2A, CIC, ARID1A, MET, and SMARCA4	2.7	1	n/a
WT	LN	BRAF, TERT, TP53 and CDK4	29.55	33.8	n/a
WT	LN	TP63, DDR2, ATM, MAP2K1, HNF1A, TERT	5.2	8	n/a

Supplementary Figure 10 Clinical characteristics of complementary FFPE staining cohort patients.
